# Effect of Isocaloric Meals on Postprandial Glycemic and Metabolic Markers in Type 1 Diabetes—A Randomized Crossover Trial

**DOI:** 10.3390/nu15143092

**Published:** 2023-07-10

**Authors:** Afroditi Alexandra Barouti, Anneli Björklund, Sergiu Bogdan Catrina, Kerstin Brismar, Neda Rajamand Ekberg

**Affiliations:** 1 Department of Molecular Medicine and Surgery, Karolinska Institute, 17176 Stockholm, Swedenneda.ekberg@ki.se (N.R.E.); 2 Center for Diabetes, Academic Specialist Center, 11365 Stockholm, Sweden

**Keywords:** type 1 diabetes, postprandial glycemia, triglycerides, isocaloric meals, macronutrients, glucagon

## Abstract

The aim of this study was to assess the effect of four isocaloric meals with different macronutrient compositions on postprandial blood glucose, lipids, and glucagon in adults with type 1 diabetes (T1D). Seventeen subjects tested four isocaloric meals in a randomized crossover design. The meal compositions were as follows: high-carbohydrate (HC); high-carbohydrate with extra fiber (HC-fiber); low-carbohydrate high-protein (HP); and low-carbohydrate high-fat (HF). Blood glucose and lipid measurements were collected up to 4 h and glucagon up to 3 h postprandially. Mean postprandial glucose excursions were lower after the HP compared to the HC (*p* = 0.036) and HC-fiber meals (*p* = 0.002). There were no differences in mean glucose excursions after the HF meal compared to the HC and HP meals. The HF meal resulted in higher triglyceride excursions compared to the HP meal (*p* < 0.001) but not compared to the HC or HC-fiber meals. Glucagon excursions were higher at 180 min after the HP meal compared to the HC and HF meals. In conclusion, the low-carbohydrate HP meal showed the most favorable glycemic and metabolic effects during a 4 h postprandial period in subjects with T1D.

## 1. Introduction

Postprandial glycemia is an important determinant of long-term glycemic control (HbA1c) and is independently associated with the risk of developing complications, including cardiovascular disease (CVD), in people with diabetes [1,2]. Achieving optimal postprandial glucose control is especially challenging for those with type 1 diabetes (T1D) [3].

Carbohydrate intake affects postprandial glucose levels more than any other dietary component/factor [4]. Accumulating evidence shows that the fat and protein contents of meals also affect postprandial glucose levels. Specifically, adding different amounts of protein and/or fat to meals with identical carbohydrate content leads to higher insulin requirements in people with T1D and higher postprandial glucose excursions, especially in the late postprandial state [5,6]. Several factors contribute to this, including postprandial differences in the secretion of glucagon, conversion of amino acids to glucose (gluconeogenesis), and delayed gastric emptying [6,7]. Previous studies have focused primarily on comparing meals with the same carbohydrate amount and varying energy and other macronutrient content. However, it is unclear how changing the macronutrient composition of isocaloric meals, i.e., meals with the same energy content, will affect postprandial glycemia in T1D.

Postprandial lipemia is also independently associated with the risk of developing CVD and diabetic complications [8,9,10]. There is, however, very limited research on how different mixed meals affect postprandial blood lipids in T1D [11] and whether meals with a favorable effect on glucose could impact lipemia negatively.

The aim of this study was to investigate how four isocaloric meals with different macronutrient compositions affect postprandial blood glucose, lipid, and glucagon levels in adults with T1D. We also investigated the effect of the same meals in a control group of healthy subjects as well as the differences between the two groups.

## 2. Materials and Methods

### 2.1. Study Design

The present study was conducted at the Department of Endocrinology, Metabolism, and Diabetes at Karolinska University Hospital in Stockholm, Sweden, between 2010 and 2015. Subjects with T1D were recruited through advertisements to participate in the study of four isocaloric meals with different macronutrient compositions. A control group of healthy subjects was also recruited as previously described [12]. The study had a randomized crossover design. Postprandial markers of glycemic and metabolic control as well as hormonal and self-reported psychometric responses were measured. The study was conducted according to the guidelines of the Declaration of Helsinki and was approved by the Regional Ethics Review Board of Stockholm (Clinical Trials.gov registration number: NCT02544568).

### 2.2. Study Participants

The inclusion criteria were age ≥20 and ≤75 years and a disease duration of ≤40 years. The exclusion criteria were pregnancy, heart failure (NYHA class III-IV), renal failure (P-creatinine >200 µmol/L), and liver disease (P-ALAT ≥ 2 µKat/L). A total of 18 subjects with T1D and 21 healthy control subjects were recruited. Written informed consent was obtained from all participants before study enrollment. One participant with T1D was excluded from further analysis due to completing only one test meal, and, therefore, the analyses were performed on 17 participants with T1D.

### 2.3. Study Procedures

During a first screening visit, participants’ fasting blood, urine, and anthropometrical measurements were taken. After screening and before the first test meal occasion, they were instructed to complete a 3-day diary including capillary blood glucose measurements when fasting in the morning, before and 2 h after each meal, and before bedtime. This was used to optimize insulin doses for the T1D group. All participants used rapid-acting insulin analogs for their meal doses (35% insulin lispro and 65% insulin aspart). Capillary blood glucose measurements (at least seven) were also recorded for the 24 h before each testing occasion. Each participant was assigned to a random sequence of meals, which was generated by a computer software (http://www.randomization.com (accessed on 25 June 2007)). The wash-out period between the meals was at least one month. Participants were instructed to consume their usual diet during the study period.

On the day of the test meal, a standardized breakfast (ca. 420–440 kcal, 58% of energy intake from carbohydrates, 22% from protein, and 20% from fat) was eaten four hours before the test meals to prevent differences due to any remaining breakfast effects. In the event of hypoglycemia during the 4 h period before the meal or during testing, the participants were instructed to correct it as per usual management, with dextrose tablets (3 g carbohydrates/tablet) being the first-line treatment recommendation. The amount of food consumed for the correction of hypoglycemia, which did not exceed 20 g carbohydrates, was noted, and then used as a covariate in the statistical analysis models (see statistical analysis).

The test meals were served at approximately 11.00 am. Participants were instructed to abstain from any physical activity between breakfast and the test meal. The recommended meal insulin doses were calculated based on carbohydrate counting using a standard insulin-to-carbohydrate ratio (ICR); if needed, participants took a small correction dose for hyperglycemia based on their insulin sensitivity factor (ISF) and adjusted the recommended dose according to their personal experience of similar meals. The postprandial testing period was four hours, during which participants were seated and engaged only in sedentary activities.

### 2.4. Test Meals

The macronutrient composition of each meal is presented in Table 1 and detailed nutritional and food content is available in the Supplementary Materials. The four isocaloric meals (620 kcal, ±20 kcal) were: a high carbohydrate reference meal (HC) based on the Nordic Nutrition Recommendations 2004, updated 2012 [13,14]; a high carbohydrate meal with approximately 35% extra fiber (HC-fiber); a low carbohydrate–high protein meal (HP); and a low carbohydrate–high-fat meal (HF). The test meals were traditional Swedish dishes and consisted of red meat (roast beef or entrecote), potatoes (boiled or fried), different vegetables and legumes, a starter for the HP meal (avocado and shrimps), or a dessert for the HC and HC-fiber meals (berries with ice cream/whipped cream). The meals were prepared under controlled conditions using kitchen scales for weighing ingredients and were served at a restaurant at Karolinska University Hospital, where the participants ate in a real-life dining environment, in each other’s company.

### 2.5. Assessment of Outcomes

The primary endpoint was plasma glucose excursions during the 4 h postprandial period. Secondary outcomes were postprandial excursions of triglycerides, total cholesterol, HDL-cholesterol, and LDL-cholesterol. Glucagon excursions were also analyzed in a subsample of T1D participants (the first 12 participants included in the study) and in all healthy controls. Blood samples were drawn with the use of a peripheral venous catheter at −30 and −5 min (before meal consumption) and every 30 min up to 240 min postprandially for glucose (and insulin concentrations for the control group). Lipids were assessed at −30 min and every 60 min up to 240 min postprandially, and glucagon at −30, 60, and 180 min. Blood samples that were not analyzed directly were stored at −80 °C. All laboratory analyses were performed blinded to the assigned test meal.

Plasma glucose, lipid profile, and screening biochemistry (HbA1c, high-sensitivity CRP, creatinine, and liver enzymes) were analyzed according to routine methods at Karolinska University Hospital (https://www.karolinska.se/for-vardgivare/karolinska-universitetslaboratoriet/ (accessed on 29 September 2009)). High-sensitivity CRP (hs-CRP) was also analyzed on each day of testing for the T1D group to control for temporary changes in insulin sensitivity due to underlying infection. One subject with T1D had hs-CRP > 10 mg/L on one test occasion and consequently the outcome values of that test meal were excluded. HbA1c was determined using the MonoS method, Unimate (Roche Diagnostics, Basel, Switzerland) in the first years of the study; these values were recalculated to the IFCC standard (mmol/mol): IFCC = (10.11 × Mono-S) − 8.94. Glucagon was measured using the RIA-kit GL-32K (Millipore, MA, USA) and insulin using ELISA (DAKO, Agilent Technologies, Glostrup, Denmark).

### 2.6. Statistical Analysis

Descriptive summaries are presented as numbers (percentages) or medians (interquartile range, IQR). Mean postprandial excursion (calculated as the postprandial value minus the preprandial value), peak postprandial excursion, and time to peak excursion were assessed for glucose and triglycerides. For glucagon, only mean postprandial excursions were analyzed, since there were just two postprandial time points available.

Linear mixed-effects models were used to test for differences in outcomes between the four test meals within each group. In the T1D group, time, meal-time interaction, BMI, meal insulin dose, extra food eaten due to hypoglycemia, the respective preprandial outcome levels, and hs-CRP levels on the day of testing were added as fixed effects in the model and participants were treated as random effects. In the control group, the covariates used were time, meal-time interaction, BMI, extra/less food eaten, respective preprandial outcome levels, and insulin concentrations. The covariates were chosen a priori to control for within-subject factors, other than the test meals, that could affect the postprandial glycemic and metabolic responses. In comparisons between the T1D and control group, the models included age, sex, extra food eaten, BMI, and respective preprandial outcome levels as between-subject fixed effects. Time was modeled using restricted cubic splines with four knots for outcomes with more than three measurement time points. For three or fewer measurement time points, time was included linearly. The robust sandwich estimator was used to estimate standard errors, which is robust to misspecifications of the variance–covariance model. The number of hypoglycemic events was compared between meals using the chi-squared test, while the odds of postprandial hypoglycemia were estimated with multilevel mixed-effects logistic regression.

*p*-values < 0.05 were considered significant. In the case of multiple comparisons between the T1D and control group over test meals, the Bonferroni correction was used, whereas for comparisons between groups a *p*-value ≤ 0.0125 (0.05/4 meals) was considered significant (Bonferroni adjustment for multiplicity). This was an exploratory study at the time it was performed, and therefore power calculations were not carried out. Analyses were performed with STATA/IC Version 16.1 (StataCorp, College Station, TX, USA).

## 3. Results

The baseline characteristics of the T1D and control group are presented in Table 2. The two groups had similar characteristics except for HbA1c, HDL cholesterol, and eGFR. Specifically, median eGFR was lower in the T1D group but none of the participants had moderate or severe loss of kidney function (min–max eGFR: 60–103 mL/min/1.73m^2^; min–max P-creatinine: 48–99 µmol/L). The median insulin doses (IQR) taken by the T1D group were 6 IU for the HC (4–8 IU) and HC fiber meals (5–8 IU) and 4 IU for the HP (4–6 IU) and HF meals (4–5 IU) (*p* = 0.019).

### 3.1. Postprandial Glucose Excursions

**Figure 1:**
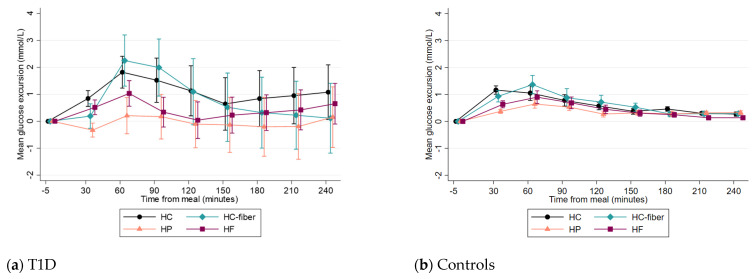
Mean glucose excursions (±SEM) by meal type from −5 to 240 min in the T1D group (**a**) and control group (**b**).

In the T1D group, mean glucose levels increased after the intake of all meals except for the HP meal (Table 3), for which a significant meal-time interaction was detected (*p* ≤ 0.05). Glucose levels reached the respective preprandial values within 120 min after the HP and HF meals (Figure 1a). At 240 min, however, only the HP meal maintained significantly lower glucose excursions compared to the HC-fiber meal (*p* = 0.003), and no other differences between meals were observed. Peak glucose excursions from baseline and time to peak were not significantly different between meals, after pairwise comparisons (Table 3). Postprandial glucose excursions per individual and meal are shown in Supplementary Materials, Figure S1a–d.

In total, 19 hypoglycemic events (2.1–3.8 mmol/L) occurred in the postprandial phase of the study, none of which was severe (Table 3). The number of events did not differ between meals (*p* = 0.23) but the odds of hypoglycemia were significantly lower after consuming the HC-fiber (odds ratio 0.07 (95% CI 0.03–0.16)), HP (0.28 (0.15–0.52)) and HC meals (0.18 (0.08–0.38)) compared to the HF meal. The timing of the hypoglycemic events differed between meals, and the majority occurred at 180 min after the HC meal, at 240 min after the HC-fiber meal, at 30, 90, and 210 min after the HP meal, and at 30 and 90 min after the HF meal.

In the control group, peak glucose excursions were lower after the HP meal compared to all other meals (*p* ≤ 0.016) (Table 3). Comparisons between the two groups showed that peak glucose excursions were higher in the T1D group after all meals compared to the control group (*p* ≤ 0.002).

### 3.2. Postprandial Lipid Excursions

**Figure 2:**
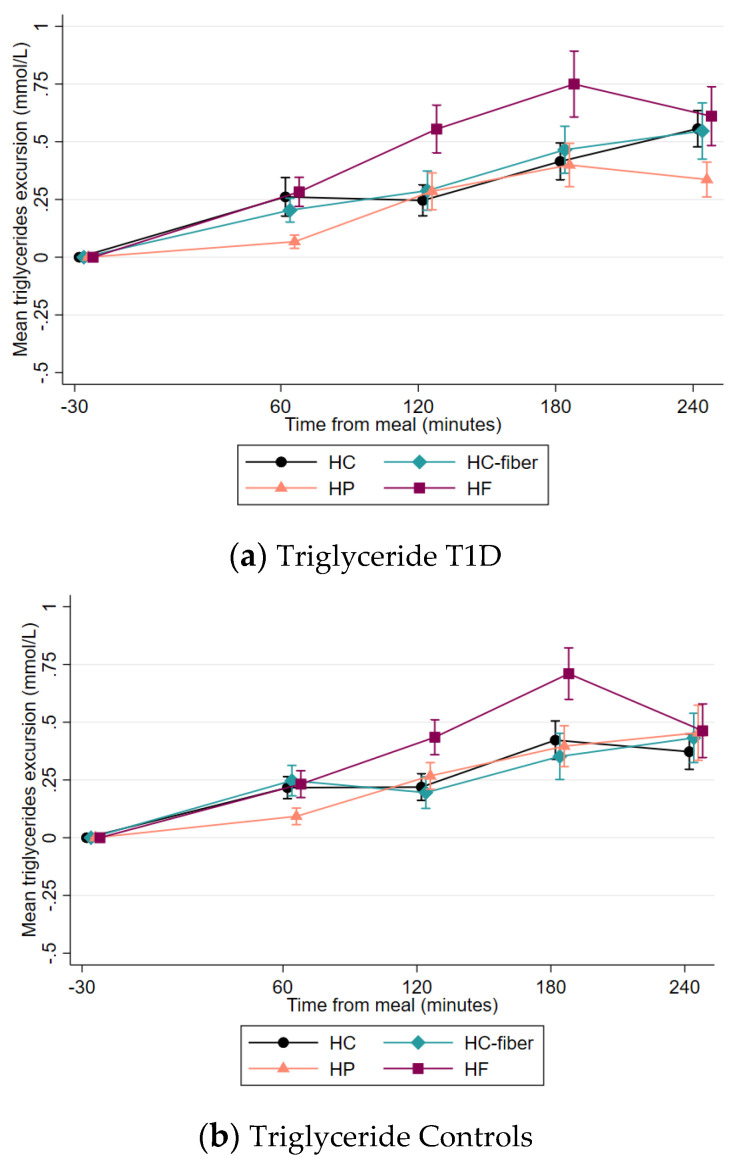

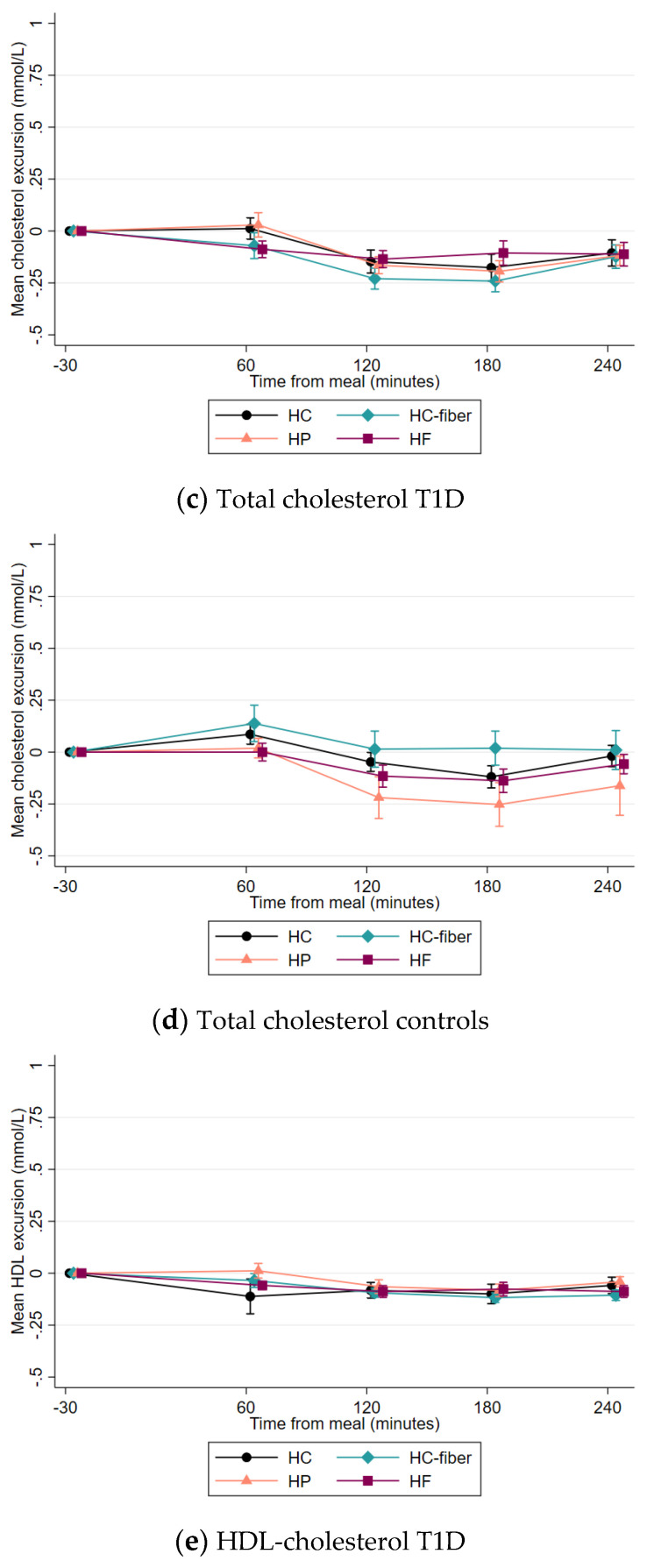

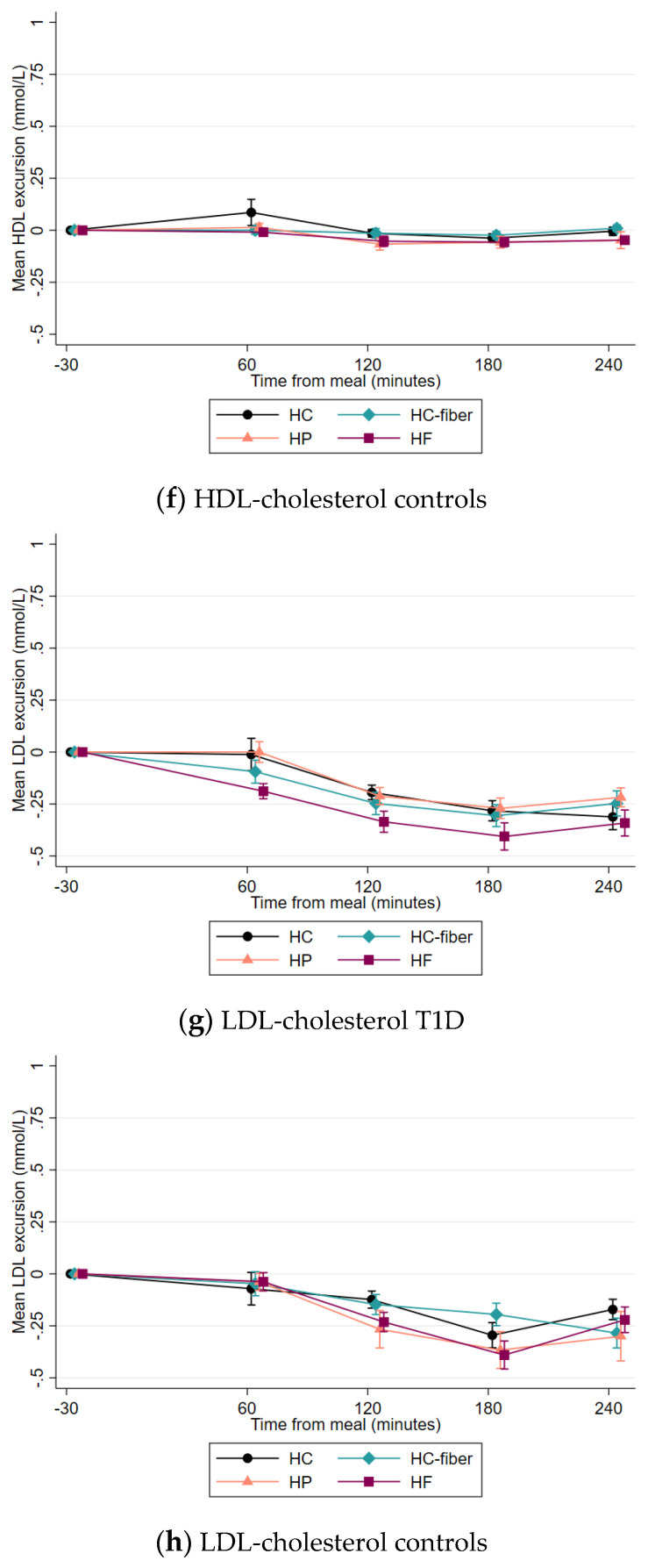
Mean lipid excursions (±SEM) by meal type from −5 to 240 min in the T1D group (**a**,**c**,**e**,**g**) and control group (**b**,**d**,**f**,**h**).

In the T1D group, the HF meal resulted in higher mean postprandial excursion, and peak excursion of triglycerides compared to the HP meal (*p* < 0.001) but there were no differences compared to the HC and HC-fiber meals (Table 3). At 240 min, both the HC and the HF meal led to significantly higher triglycerides compared to the HP meal (*p* ≤ 0.010). Postprandial excursions of total cholesterol and HDL-cholesterol were not significantly different between meals (Figure 2c,e). LDL-cholesterol decreased after all meals (Figure 2g) but on average 0.1 mmol/L more after the HF than the HP meal (*p* = 0.003).

In the control group, postprandial triglyceride excursions had a similar trend to that observed in the T1D group (Figure 2b), with the HF meal resulting in higher mean excursions compared to the HP meal (Table 3). At 240 min postprandially, there were no longer differences in triglyceride excursions between meals (in contrast to the T1D group). Postprandial excursions of total cholesterol, HDL-, and LDL-cholesterol did not differ significantly between meals in the control group (Figure 2d,f,h). Comparisons of postprandial lipid excursions between the T1D and control group per meal showed no overall significant differences.

### 3.3. Postprandial Glucagon Excursions

**Figure 3:**
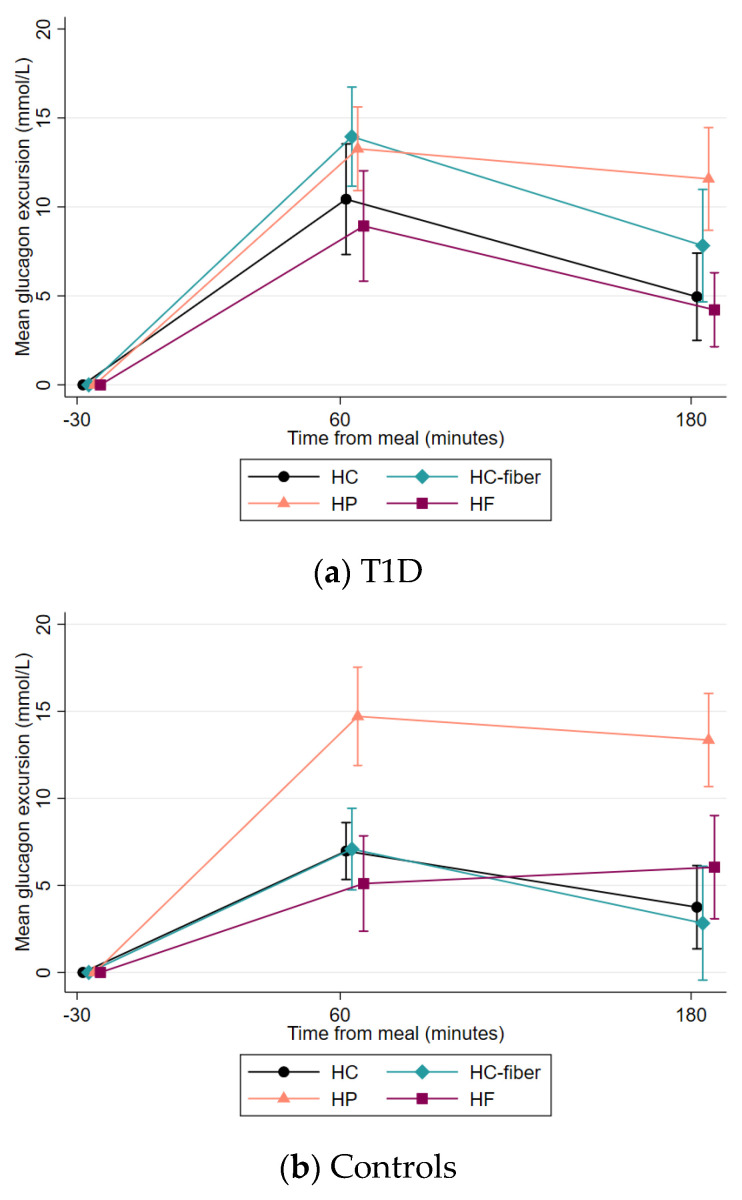
Mean glucagon excursions (±SEM) by meal type from −5 to 180 min in the T1D group (**a**) and control group (**b**).

In the T1D group, glucagon concentrations increased after the consumption of all four meals at 60 min and then decreased for all meals except for the HP meal, resulting in significant differences with the HC and HF meals at 180 min (*p* ≤ 0.002) (Figure 3a). The control group had higher glucagon excursions after the HP meal at both measurement points, 60 and 180 min, compared to all other meals (*p* ≤ 0.002). There were no significant differences when comparing the postprandial glucagon excursions of the T1D group with the control group after each meal.

## 4. Discussion

In this randomized crossover study in people with T1D, overall postprandial glucose excursions were lower after the low-carbohydrate HP meal compared to the isocaloric high-carbohydrate meals with or without extra fiber. The HF meal increased postprandial triglyceride levels significantly more compared to the HP meal but not compared to the HC or HC-fiber meals. Glucagon excursions were higher 180 min after the HP meal compared to the HC and HF meals. In comparison with the control group, the T1D group had significantly higher peak glucose excursions after all meals.

In contrast to previous studies, neither the HP nor the HF meal resulted in higher postprandial glycemia in the later postprandial phase (120–240 min) compared to the high-carbohydrate meals, which can be attributed to our isocaloric study design. In particular, previous studies in subjects with T1D have mainly compared meals and test drinks that had the same carbohydrate amount but differed in energy, fat, and/or protein content [15,16,17,18,19,20,21]. Most of them found that the addition of either fat or protein to a carbohydrate-only meal/drink independently increases and delays postprandial glycemia from 3 h onward and their combined consumption has an additive impact on glucose levels [5,17,18,21]. Part of the delayed hyperglycemia can be explained by the delayed gastric emptying after the addition of fat and the conversion of amino acids to glucose (gluconeogenesis) [7,19]. However, when mixed meals are consumed in real life, the three macronutrients substitute each other, which makes isocaloric meals highly relevant. In our study, keeping the energy content of the meals stable while changing the proportions of the macronutrients towards a low carbohydrate composition (even with significant increases in the absolute amounts of fat or protein), did not lead to higher blood glucose excursions during the 4 h postprandial period.

There was no significant difference in postprandial glucose excursions between the HP and HF meal in this study, neither in the earlier nor in the later postprandial phase. However, other studies have shown that glucose levels increased significantly in the later postprandial phase after HP meals compared to isocaloric HF meals of equal carbohydrate content in T1D [22,23,24]. One reason for the different findings could be that our study’s postprandial period was shorter compared to these studies, in which higher glucose excursions appeared at 180–300 min [7,23,24] or even later [22]. Furthermore, a positive dose-dependent relationship has been reported between the amounts of protein or fat consumed in carbohydrate-containing meals and late postprandial glucose excursions [17,25]. It is possible that the absolute differences in protein and fat amounts between the HP and HF meal in this study were not large enough to induce a higher late postprandial response after the HP meal. Future studies could further investigate whether there is an optimal threshold for increasing protein while decreasing fat intake in carbohydrate-containing meals, and vice versa, to prevent late postprandial hyperglycemia.

Unexpectedly, the HC-fiber meal did not result in lower postprandial glycemia compared to the standard HC meal in this study, neither in the T1D group nor in the healthy controls, as seen previously in other studies [5]. The lack of postprandial glycemic differences between the two HC meals could be due to the already high-fiber content of the standard HC meal in our study (43% of the daily recommended intake of 25–35 g in Sweden), which may have hindered comparisons with the HC-fiber meal. In addition, we could hypothesize that differences in meal insulin doses in the T1D group, although not statistically significant, may have partly played a role, as some subjects took less insulin for the HC fiber than the HC meal despite their almost equal carbohydrate content.

Glucagon concentrations remained significantly higher after the HP meal compared to the HC and HF meals at 180 min, in agreement with previous studies [26]. Despite this, glucose levels did not increase significantly at 180–240 min after the HP meal compared to the other meals. The rise in circulating glucagon after the consumption of HP meals [7] has been proposed as a measure for preventing nocturnal hypoglycemia in people with T1D [22,27], and notably the HP meal also resulted in fewer hypoglycemic events than the HF meal in our study. Interestingly, triglyceride excursions were lower 180–240 min after the HP meal compared to the HC and HF meals, while glucagon excursions were significantly higher at 180 min. Glucagon has been proposed to have a role in lipid metabolism, including the stimulation of hepatic beta-oxidation and inhibition of hepatic lipogenesis, but further research is needed to understand the postprandial effects [28].

Postprandial hyperlipidemia is an independent risk factor for CVD and is influenced by many dietary components; however, studies investigating the effect of different meals on postprandial lipemia in people with T1D are scarce. In our study, there was no difference in postprandial lipids between the isocaloric HC meals and the HF meal. The HC meals included higher amounts of simple sugars, while the HF meal had a higher amount of total fat, which are the dietary factors that have been found to increase postprandial triglycerides the most [29]. Moreover, the HF meal, with relatively low SFA content, increased postprandial triglycerides significantly more than the isocaloric lower-in-fat HP meal in both the T1D and control groups. Similar findings were reported in a previous study where two high-fat meals (one high in MUFAs and one high in SFAs) resulted in higher iAUC for triglycerides compared to a meal with identical carbohydrate but lower fat and energy contents [19]. Finally, there was a modest decrease in postprandial LDL-cholesterol after the consumption of all meals in this study, which has also been reported in healthy subjects [30].

The acute postprandial glycemic and metabolic responses after isocaloric meals could be linked to some of the long-term health effects of different diets and dietary patterns. In our study, we found more favorable glycemic effects after the HP and HF meals, which reflect the macronutrient composition of low carbohydrate diets (LCDs). LCDs have been found to reduce HbA1c in people with T2D [31] and T1D [32] and decrease time spent in hypoglycemia and glycemic variability in people with T1D [33]. Moreover, the higher MUFA content of our HP and HF meals resembles Mediterranean-style diets, which have shown beneficial long-term effects on CVD risk factors in T2D [31]. Currently, no specific diet is considered superior to others in the management of T2D, with the ADA and European guidelines recommending a variety of eating patterns with different macronutrient compositions [31,34,35]. However, the available research on T1D is insufficient to provide similar recommendations [31,35].

Regarding protein intake, evidence suggests that some people with diabetes and eGFR > 60 mL/mil per 1.73 m^2^ may have higher protein requirements than the general public. Specifically, those aged 65 years or older may need protein intakes of 15–20% of total energy to prevent loss of muscle mass and sarcopenia, while those on weight loss diets may benefit from even higher intakes (23–32% of total energy) [34]. However, there are also concerns about worsening renal function with high dietary protein intake, especially in individuals with pre-existing kidney disease, as shown in some observational studies and short-term trials [36]. Notably, a recent meta-analysis of people with diabetes has not found an acceleration of the loss of kidney function with higher protein intakes [37]. Based on the conflicting evidence available, high protein intakes should be considered with caution in people with pre-existing kidney disease.

This study had several strengths. It was crossover and allowed comparisons both within and between subjects. It investigated four different meals and had an isocaloric design. The meals studied were proper restaurant-cooked dishes commonly eaten in Sweden instead of test drinks/meals and the study simulated a real-life dining environment. Furthermore, the control group of healthy subjects that was used for comparisons with the T1D group was well-matched and did not differ in most baseline characteristics; HbA1c was expectedly lower, and the lower HDL-cholesterol levels could be explained by the higher number of men in the control group. Adjustments for multiple comparisons between the T1D and control group over test meals were also included.

There were, however, some limitations. First, some subjects experienced hypoglycemia before the test meals and needed to compensate with extra dextrose or food. Nevertheless, we tried to correct for this by adding covariables in the linear mixed effects models. Secondly, the 4 h duration of the postprandial testing period in this study may be short, as late postprandial glycemia has been investigated between 3 and 5–6 h in previous studies [17,18,19,23,24]. However, the specific duration was chosen to resemble real-life eating conditions, since the next meal or snack would be usually consumed after or within this timeframe. Moreover, continuous glucose monitoring systems were not commonly used at the time this study was conducted, and therefore information on time in range, time spent in hypoglycemia, or hyperglycemia that are nowadays important measures of postprandial and total glycemic control, is not available.

In conclusion, comparisons of real-life isocaloric meals showed that a high-protein meal with lower carbohydrate content (43 g, 33% EI) had more favorable glycemic effects than isocaloric high-carbohydrate meals (75–79 g, 53–54% EI) during a 4 h postprandial period in people with T1D. The high-protein meal also resulted in fewer hypoglycemic events and lower postprandial triglyceride excursions compared to the isocaloric high-fat meal, and, therefore, its macronutrient composition may be recommended for improving postprandial glycemic and metabolic control in people with T1D, without pre-existing kidney disease. Further studies with isocaloric meals containing all three macronutrients are needed to better understand the respective contributions of fat and protein to postprandial glycemia and lipemia in T1D.

## Figures and Tables

**Table 1:** Meal composition.

Meals	HC	HC-Fiber	HP	HF
Energy (kcal)	638	640	600	606
Carbohydrates (% EI)	54	53	33	32
Fiber (g)	13	18	11	11
Protein (% EI)	18	22	36	18
Fat (% EI)	28	25	31	50
SFA (% EI)	10	6	6	12
MUFA (% EI)	12	12	18	28
PUFA (% EI)	4	5	5	7

HC, high-carbohydrate; HC-fiber, high-carbohydrate with extra fiber; HP, low carbohydrate–high protein; HF, low carbohydrate–high fat; % EI, percentage of energy intake.

**Table 2:** Baseline characteristics of study participants.

	T1D Group (*n* = 17)	Control Group (*n* = 21)	*p* ^a^
Sex			0.393
Females	12 (71%)	12 (57%)	
Males	5 (29%)	9 (43%)	
Age (years)	64 (39, 67)	57 (42, 64)	0.509
Diabetes duration (years)	16 (10, 30)	-	-
BMI (kg/m^2^)	24.7 (22.5, 25.4)	23.5 (21.2, 26.8)	0.386
Waist (cm)	85.5 (80.3, 93.3)	85 (78, 90)	0.452
Systolic blood pressure (mmHg)	125 (117, 141)	130 (120, 143) ^b^	0.562
Diastolic blood pressure (mmHg)	75 (71, 81)	81 (74, 88) ^b^	0.143
Diabetes treatment			-
Multiple daily injections	14 (82%)	-	
Insulin pump	3 (18%)	-	
Total daily insulin (TDI) dose (IU)	30 (25, 51)	-	-
% TDI as basal insulin	51 (38, 55)	-	-
Smoking			0.935
Non-smoker	12 (71%)	14 (67%)	
Former smoker	4 (23%)	6 (29%)	
Current smoker	1 (6%)	1 (5%)	
Current medicines			
Antihypertensives	5 (29%)	2 (10%)	0.116
Lipid-lowering agents	6 (35%)	3 (14%)	0.130
Retinopathy	8 (47%)	-	-
Neuropathy	3 (18%)	-	-
Biochemistry			
HbA1c (mmol/mol)	58 (54, 65)	37 (36, 39)	<0.001
Triglycerides (mmol/L)	0.73 (0.64, 0.87)	0.76 (0.52, 0.91)	0.803
Total cholesterol (mmol/L)	4.8 (4.6, 5.5)	5.2 (4.2, 5.3)	0.780
HDL-cholesterol (mmol/L)	2.0 (1.5, 2.4)	1.5 (1.3, 1.9)	0.031
LDL-cholesterol (mmol/L)	2.5 (2.3, 2.9)	3.3 (2.4, 3.6)	0.361
hs-CRP (mg/L)	1 (0.48, 1.9)	0.5 (0.23, 0.98)	0.120
e-GFR (mL/min/1.73 m^2^)	74 (68, 91)	90 (79, 103)	0.029

Data are presented as numbers (percentages) or medians (interquartile range). ^a^ *p*-values were calculated by the Mann–Whitney U test for continuous variables and the Pearson chi-square test for categorical variables. ^b^ One missing value.

**Table 3:** Comparisons of glycemic and metabolic outcomes (mean (SD)) by meal type presented separately for the T1D and the control group.

	T1D Group (*n* = 17 *)				Control Group (*n* = 21)			
	HC	HC-Fiber	HP	HF	HC	HC-Fiber	HP	HF
**Glucose**								
Preprandial level (mmol/L)	5.8 (3.0)	8.0 (4.0)	7.0 (3.8)	6.9 (3.8)	4.9 (0.4)	4.9 (0.4)	4.9 (0.3)	5.0 (0.4)
2 h postprandial excursion (mmol/L)	1.1 (3.8)	1.1 (5.1)	−0.1 (3.6) ^a b^	0.0 (2.8) ^b^	0.6 (0.6)	0.7 (1.1)	0.3 (0.5)	0.5 (0.7)
Mean postprandial excursion (mmol/L)	1.1 (3.5)	0.8 (4.6)	−0.5 (3.7) ^a b^	0.4 (2.5)	0.6 (0.8)	0.7 (1.0)	0.4 (0.5)	0.4 (0.7)
Peak excursion (mmol/L)	3.5 (2.7)	2.9 (3.8)	2.1 (2.8)	2.3 (2.0)	1.6 (1.0)	1.7 (1.4)	1.0 (0.4) ^a b d^	1.3 (0.9)
Time to peak excursion (min)	113 (85)	74 (34)	132 (91)	129 (90)	66 (61)	66 (49)	79 (45)	86 (56)
Hypoglycemic events ^±^	6 (35%)	2 (12%)	4 (24%)	7 (41%)	-	-	-	-
**Triglycerides**								
Preprandial levels (mmol/L)	0.7 (0.3)	0.8 (0.3)	0.9 (0.5)	0.7 (0.3)	0.9 (0.4)	1.0 (0.5)	0.9 (0.4)	0.8 (0.4)
Mean postprandial excursion (mmol/L)	0.4 (0.3)	0.4 (0.4)	0.3 (0.3)	0.5 (0.5) ^c^	0.3 (0.3)	0.3 (0.4)	0.3 (0.4)	0.5 (0.4) ^c^
Peak excursion (mmol/L)	0.7 (0.3)	0.6 (0.5)	0.5 (0.4)	0.9 (0.5) ^c^	0.5 (0.4)	0.6 (0.5)	0.6 (0.5)	0.8 (0.5)
Time to peak excursion (min)	173 (76)	187 (67)	187 (47)	169 (61)	157 (64)	154 (75)	177 (67)	149 (52)
**Glucagon**								
Preprandial levels (pg/mL)	57.3 (7.2)	56.0 (7.3)	59.0 (9.6)	61.2 (11.7) ^b^	82.0 (20.0)	80.0 (24.9)	91.3 (23.1)	78.8 (20.6)
Mean excursion (pg/mL)	7.7 (9.9)	10.9 (10.6)	12.4 (9.0) ^a^	6.6 (9.3)	5.4 (9.4)	5.0 (13.1)	14.0 (12.5) ^a b d^	5.6 (12.9)

* *n* = 17 for all analyses except for glucagon where analyses were performed only in 12 subjects with T1D. ^±^ Hypoglycemic events (2.1–3.8 mmol/L) are presented as number of events per meal (%). Linear mixed-effects models were used to test for differences in continuous outcomes between meals within each group. The fixed effects were time, meal–time interaction, BMI, meal insulin dose, extra food eaten due to hypoglycemia, the respective preprandial outcome levels, and hs-CRP levels on the day of testing in the T1D group; and time, meal–time interaction, BMI, insulin levels, the respective preprandial outcome levels, and extra/less food eaten at meals in the control group. Participants were treated as random effects. Significant differences (*p* ≤ 0.05) after pairwise comparisons are presented as follows: ^a^ significantly different from the HC meal; ^b^ significantly different from the HC-fiber meal; ^c^ significantly different from the HP meal; and ^d^ significantly different from the HF meal.

## Data Availability

The data presented in this study are available upon request from the corresponding author.

## Supplementary Materials

The following supporting information can be downloaded at: https://www.mdpi.com/article/10.3390/nu15143092/s1, Meal description and nutritional content; Figure S1: Postprandial glucose excursions per subject and meal.
