# Supplementary material for: Effect of Isocaloric Meals on Postprandial Glycemic and Metabolic Markers in Type 1 Diabetes—A Randomized Crossover Trial
Source: Nutrients. 2023 Jul 10;15(14):3092. doi: 10.3390/nu15143092 (PMC10386239; doi:10.3390/nu15143092)
Supplement: Supplementary file 1 [file nutrients-15-03092-s001.zip › nutrients-2474703-supplementary.pdf]

Article: Effect of meal composition on glycemic and metabolic markers in type 1 diabetes – a randomized crossover trial

Afroditi Alexandra Barouti [1 2] *, Anneli Björklund [1 2], Sergiu Bogdan Catrina [1 2], Kerstin Brismar [1] and Neda Rajamand Ekberg [1 2]

[1]Department of Molecular Medicine and Surgery, Karolinska Institute, Stockholm, Sweden

[2]Center for Diabetes, Academic Specialist Center, Region Stockholm, Stockholm, Sweden

* Correspondence: afroditi.barouti@ki.se

Supplementary Materials

Meal description and nutritional content:

<u>High-carbohydrate (HC) meal based on the Nordic Nutrition Recommendations</u>

Main dish: 75 g roast beef in the oven with 235 g stir-fried vegetables (carrot, broccoli, sweet corn, leek and onion), 10 g soy sauce with rapeseed oil and 160 g boiled potatoes

Dessert: 160 g blueberries and strawberries with sprinkled sugar and 20 g cream

Nutritional content:

Energy (kcal)	638
Fat (g)	20
Of which saturated (g)	7
Mono-unsaturated (g)	8
Polyunsaturated (g)	3
Carbohydrates (g)	79
Of which sugars (g)	36
Fiber (g)	13
Protein (g)	28
Salt (g)	1.1

High-carbohydrate with extra fiber (HC-fiber) meal

Main dish: 80 g roast beef in the oven with 325 g stir-fried vegetables (carrot, broccoli, haricots verts, red beans, leek and onion), 10 g soy sauce with rapeseed oil and 100 g boiled potatoes

Dessert: 150 g blueberries and strawberries with sprinkled sugar and 30 g ice cream 15%

Nutritional content:

Energy (kcal)	640
Fat (g)	18
Of which saturated (g)	5
Mono-unsaturated (g)	8
Polyunsaturated (g)	4
Carbohydrates (g)	75
Of which sugars (g)	38
Fiber (g)	18
Protein (g)	35
Salt (g)	1.6

Low carbohydrate high-protein (HP) meal

Starter: 50 g avocado with 50 g shrimps

Main dish: 150 g roast beef in the oven with 200 g stir-fried vegetables (broccoli, sweet corn, leek and onion), 10 g soy sauce with rapeseed oil and 150 g boiled potatoes

Nutritional content:

Energy (kcal)	600
Fat (g)	21
Of which saturated (g)	4
Mono-unsaturated (g)	12
Polyunsaturated (g)	3
Carbohydrates (g)	43
Of which sugars (g)	7
Fiber (g)	11
Protein (g)	54
Salt (g)	2.3

<u>Low carbohydrate high-fat (HF) meal</u>

Main dish: 75 g entrecote fried in the pan with 140 g stir-fried vegetables (broccoli, sweet corn, carrot and onion), 10 g soy sauce with rapeseed oil, 60 g avocado and 100 g fries baked in the oven

Nutritional content:

Energy (kcal)	606
Fat (g)	34
Of which saturated (g)	8
Mono-unsaturated (g)	19
Polyunsaturated (g)	5
Carbohydrates (g)	44
Of which sugars (g)	7
Fiber (g)	11
Protein (g)	27
Salt (g)	1.2

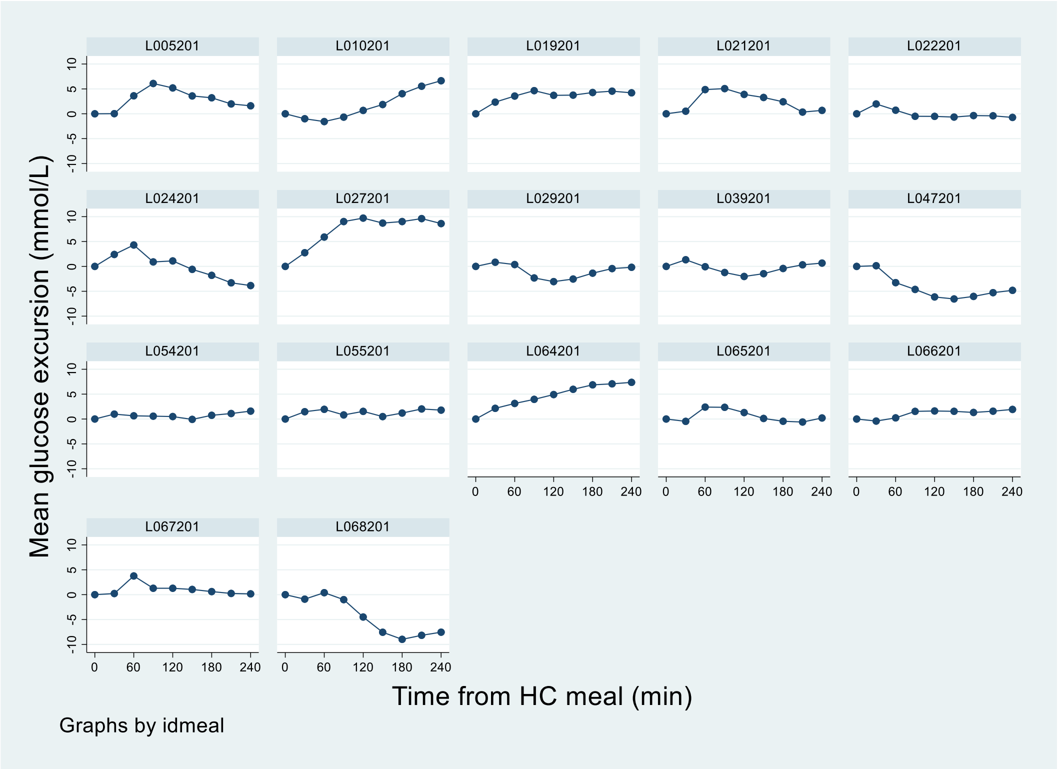

(a) HC meal

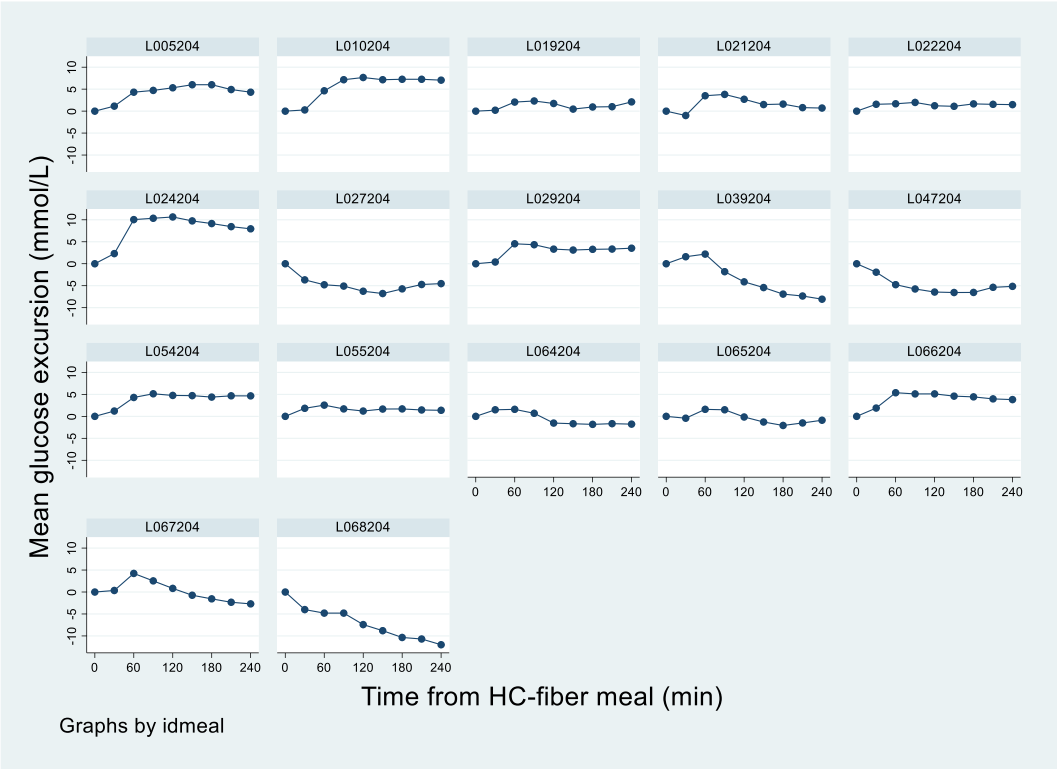

(b) HC-fiber meal

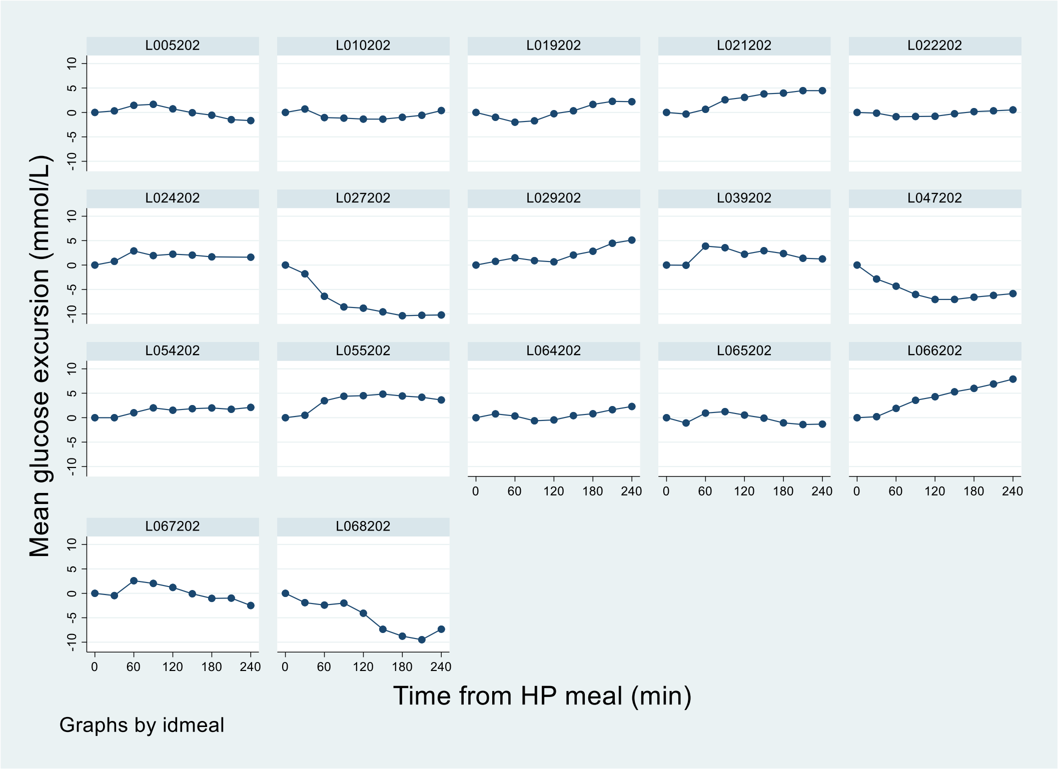

(c) HP meal

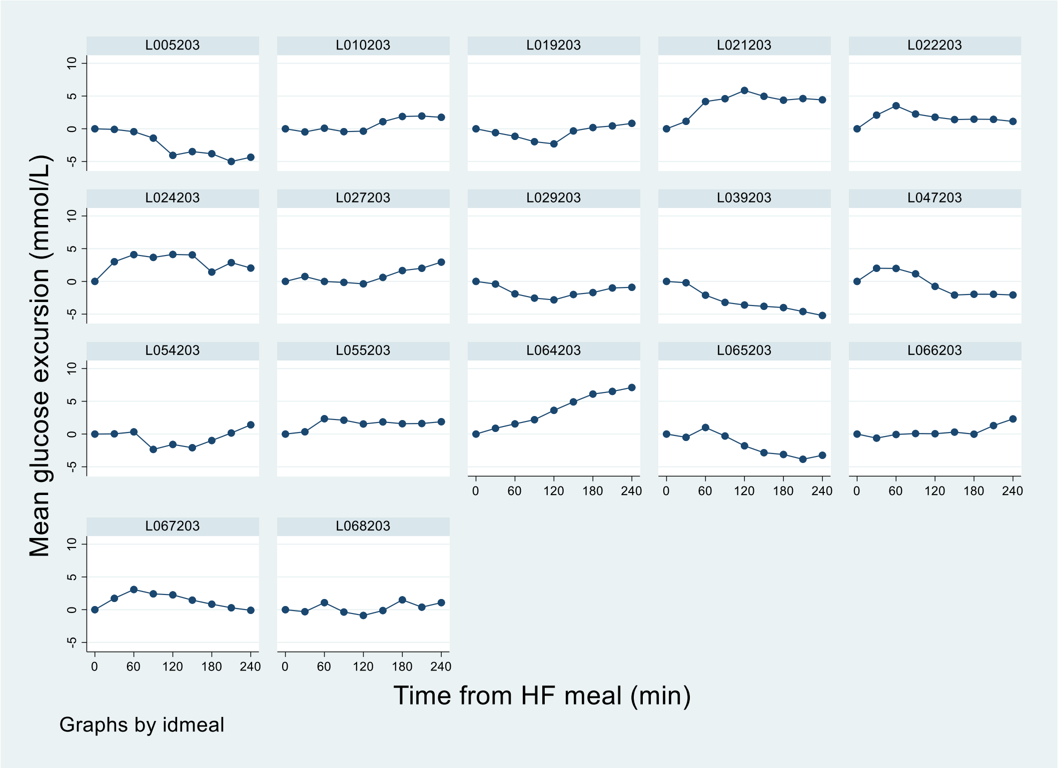

(d) HF meal

Figure S1. Postprandial glucose excursions (mmol/L) per subject and meal.
